# Surgical Performance of 3D-Printed Polyetheretherketone (PEEK) Patient-Specific Implants and Titanium Mesh in Clinically Matched Orbital Reconstruction: A Cadaveric Study

**DOI:** 10.3390/cmtr19010013

**Published:** 2026-03-02

**Authors:** Jokin Zubizarreta Oteiza, Dominik Haenggi, Yannick Simon Krieger, Lukas Schuebel, Daniel Seiler, Florian Markus Thieringer, Neha Sharma

**Affiliations:** 1 Clinic of Oral and Cranio-Maxillofacial Surgery, University Hospital Basel, 4031 Basel, Switzerland; jokin.zubizarreta@unibas.ch (J.Z.O.); florian.thieringer@usb.ch (F.M.T.); 2 Medical Additive Manufacturing Research Group (Swiss MAM), Department of Biomedical Engineering, University of Basel, 4123 Allschwil, Switzerland; 3 3D Systems, 81369 Munich, Germany; 4 Institute for Medical Engineering and Medical Informatics, University of Applied Sciences and Arts Northwestern Switzerland, 4132 Muttenz, Switzerland; daniel.seiler@fhnw.ch; 5 SingHealth Duke-NUS Radiological Sciences Academic Clinical Programme (RADSC ACP), Office of Academic Medicine, Duke-NUS Medical School, Singapore 169857, Singapore

**Keywords:** 3D printing, additive manufacturing, barium sulfate, craniomaxillofacial trauma, material extrusion, orbital fracture, polymers, radiopaque biomaterials, surgical simulation, virtual surgical planning

## Abstract

Orbital reconstruction following trauma remains challenging due to complex three-dimensional (3D) anatomy and limited surgical access. While pre-fabricated titanium mesh is standard, it requires extensive intraoperative manipulation and produces imaging artifacts. The 3D-printed polyetheretherketone (PEEK) patient-specific implants (PSIs) offer potential advantages; however, limited data exists for the acceptance of PEEK PSIs by surgeons compared to other established techniques. Fourteen surgeons performed simulated orbital reconstructions on nine cadaveric heads comparing titanium mesh and the 3D-printed PEEK PSIs. Titanium mesh was used for Class II orbital floor fractures, while the 3D-printed PEEK PSIs (native and radiopaque formulations) were used for Class IV defects. Surgeons were blinded to the PEEK formulation type. Outcomes included operative efficiency, handling characteristics, fit quality, and mechanical stability using validated 5-point Likert scales and objective timing. The 3D-printed PEEK PSIs demonstrated faster procedure times (9.5 ± 5.3 vs. 11.2 ± 5.1 min) and superior fit quality (2.00 ± 1.04 vs. 2.18 ± 0.60) and mechanical stability (1.67 ± 0.49 vs. 1.91 ± 0.54), with 100% rated stable versus 91% for the titanium mesh. Surgeons could not distinguish between the native and radiopaque PEEK formulations. Most surgeons (64.3%) preferred situation-dependent material selection. The 3D-printed PEEK PSIs demonstrated advantages in handling, fit quality, and mechanical stability for complex defects, while the titanium mesh showed a lower learning curve for simple reconstructions. Radiopaque enhancement expands PEEK’s clinical utility without compromising handling.

## 1. Introduction

Orbital reconstruction after trauma is particularly challenging because of the intricate three-dimensional (3D) structure of the orbital floor and walls, combined with restricted intraoperative visibility [1]. The current standard involves the intraoperative shaping and adaptation of pre-fabricated, stock titanium mesh implants, a process that is time-consuming, requires substantial surgical skill, and often results in suboptimal restoration [2]. Additionally, titanium mesh causes substantial imaging artefacts and beam-hardening effects that can obscure the surrounding soft tissue and complicate post-operative assessment [3], and is often associated with various complications, including implant exposure, infection, and diplopia [4]. Even when meticulously shaped, residual sharp edges can catch orbital fat or extraocular muscles during insertion or removal, increasing the risk of orbital tissue trauma [5].

The evolution toward 3D-printed patient-specific implants (PSIs) offers significant advantages over traditional approaches [6]. Point-of-care 3D printing enables streamlined workflows from computed tomography (CT) imaging to implant production, providing a precise fit and reducing operative time by eliminating manual intraoperative adaptation [7,8]. This integrated digital approach allows for pre-surgical planning and procedure rehearsal.

Polyetheretherketone (PEEK) has emerged as a promising alternative biomaterial for craniomaxillofacial (CMF) applications, offering excellent biocompatibility, chemical inertness, and imaging compatibility without artefacts that compromise diagnostic accuracy [9,10,11]. However, the inherent radiolucency of a native, unfilled PEEK biomaterial limits its orbital applications, where postoperative visualization and long-term monitoring are essential. This represents a prevalent challenge in polymer-based implantable biomaterials [12]. Incorporating barium sulfate (BaSO_4_) into PEEK provides controlled radiopacity for precise implant localization while preserving excellent soft tissue contrast and avoiding the beam hardening artefacts typical of metallic implants [13].

Despite the theoretical advantages of 3D-printed PEEK PSIs for orbital reconstruction, limited data exist on their acceptance in surgery, their handling characteristics, or their comparative performance against established prefabricated titanium mesh. Transitioning from traditional materials to innovative biomaterials requires a careful evaluation of material properties and practical surgical considerations, including ease of handling, learning curve, and surgeon confidence in achieving optimal outcomes. Additionally, successfully integrating radiopaque enhancement into PEEK formulations requires validation that the material modifications do not compromise the surgical handling characteristics.

This study aimed to assess surgeons’ evaluation of 3D-printed PEEK (native, unfilled, and BaSO4) PSIs and pre-fabricated, stock titanium mesh implants for orbital reconstruction through a comprehensive cadaveric study. Specifically, we aimed to compare the handling qualities, surgical workflow efficiency, confidence in implant positioning, and overall surgeon preference between the pre-bent titanium mesh and the 3D-printed PEEK orbital PSIs. Additionally, we sought to evaluate the learning curve for the 3D-printed PEEK orbital PSI implementation and to validate the successful integration of BaSO_4_ enhancement by assessing surgeons’ ability to distinguish between native (standard) and radiopaque PEEK formulations during surgical procedures.

## 2. Materials and Methods

### 2.1. Study Design and Participants

This study evaluated surgeons’ assessments of the conventional pre-bent stock titanium mesh and the 3D-printed PEEK PSIs (native PEEK and radiopaque barium sulfate-enhanced PEEK (BaSO_4_-PEEK) for orbital reconstruction. This comparative cadaveric study was designed as a pragmatic clinical simulation that reflects real-world fracture–implant matching rather than a controlled material-property comparison. Implant selection was intentionally matched to the clinically appropriate fracture complexity to replicate routine clinical decision-making in orbital reconstruction.

This study was conducted in accordance with institutional guidelines for cadaveric research, with ethical approval obtained from the Ethics Commission of Northwestern and Central Switzerland (EKNZ). A total of 9 fresh-frozen cadaveric heads (18 orbital sites) were obtained from Rise Labs (Benit 30A, 1043BB Amsterdam, The Netherlands) with appropriate consent for research use and prepared for orbital reconstruction procedures under standardized surgical procedures. Preoperative high-resolution computed tomography (CT) images of the cadaver heads (SOMATOM go.Now, Siemens Healthineers AG, Forchheim, Germany) were obtained to enable the design of anatomically accurate PSIs based on existing orbital anatomy.

Participants with varying levels of craniomaxillofacial (CMF) surgical training were recruited from the Clinic of Oral and Cranio-Maxillofacial Surgery at University Hospital Basel and affiliated training programs through direct invitation. The participants were informed that the study aimed to compare the surgical handling characteristics of different orbital reconstruction materials and technologies. To minimize bias, the participants were blinded to the specific hypotheses regarding material superiority and were not informed which PEEK formulation (native vs. radiopaque) they were using during each procedure. Those participants without prior orbital reconstruction experience received standardized orientation on surgical approaches and implant handling principles prior to evaluation. Demographic data were collected, including sex, age, years of surgical experience, and the number of previously performed orbital reconstructions. All participants provided informed consent. Each participant performed orbital reconstruction procedures using all materials on different randomized cadaveric models, with a comprehensive evaluation completed immediately following each procedure using a structured questionnaire. The surgeons’ identities were anonymized during data collection and analysis, per applicable privacy regulations and research ethics standards.

### 2.2. Surgical Case Complexity and Implant Assignment

This design reflects real-world clinical decision-making, where implant selection is inherently matched to fracture complexity rather than randomized across identical defect patterns. Implant evaluation was conducted using clinically appropriate fracture complexities for each material based on the Jaquiéry classification system [14]. Titanium mesh orbital reconstruction procedures were performed on simulated Class II orbital fractures (defects > 2 cm^2^ of the floor and/or medial wall within zones 1–2), representing moderate defects where manual intraoperative contouring is clinically feasible. The 3D-printed PEEK PSIs were designed to simulate Class IV orbital fractures (entire orbital floor and medial wall defects extending into the posterior third/zone 3, with a missing bony ledge), representing complex multi-walled defects in which patient-specific reconstruction provides maximum clinical benefit. This design reflects real-world clinical decision-making, where implant selection is matched to fracture complexity (Table 1).

### 2.3. Virtual Surgical Planning and Implant Fabrication

High-resolution CT imaging data (0.8 mm slice thickness) were processed using medical image processing software (Materialise Mimics Innovation Suite v26, Materialise NV, Leuven, Belgium) to create digital anatomical models of orbital anatomy for each cadaveric specimen. Anatomical biomodels were 3D-printed using material extrusion or fused filament fabrication (FFF) technology (Bambu Lab X1 Carbon, Bambu Lab, Shenzhen, China) with polylactic acid (PLA) filament (Matte Ivory White filament Bambu Lab, Shenzhen, China) as surgical planning templates.

**Figure 1:**
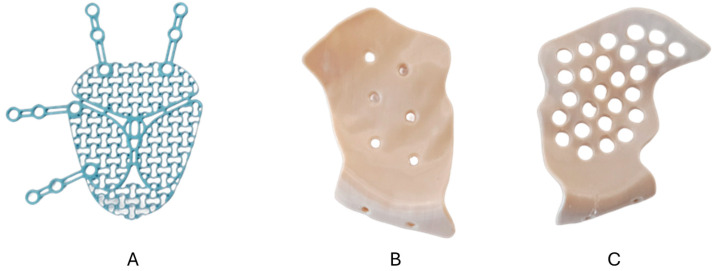
Orbital implants evaluated for surgical performance. (**A**) Pre-fabricated titanium mesh requiring intraoperative contouring. (**B**) 3D-printed patient-specific native (unfilled) PEEK implant. (**C**) 3D-printed patient-specific radiopaque PEEK (BaSO_4_-enhanced) implant.

**Figure 2:**
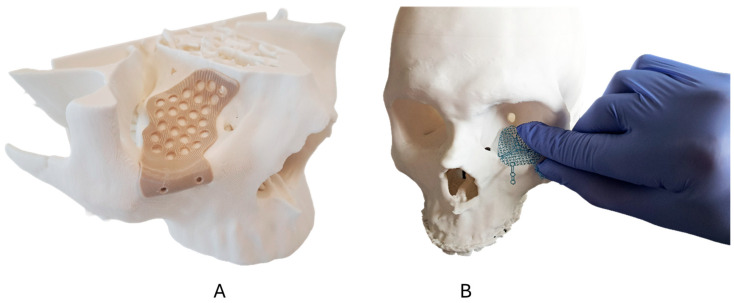
3D-printed orbital biomodels for preoperative planning. (**A**) Biomodel with patient-specific PEEK implant demonstrating pre-designed fit. (**B**) Biomodel used for titanium mesh pre-contouring to match patient-specific orbital anatomy.

Pre-fabricated stock titanium meshes (Figure 1A) (Medartis Orbital Plating System OPS 1.5, M-4440, 0.3 mm, Medartis AG, Basel, Switzerland) were pre-bent using the 3D-printed anatomical models and (Figure 2B) underwent final intraoperative adjustment (if needed) according to established surgical protocols.

The 3D-printed PEEK PSIs were designed using computer-aided design (CAD) software (Geomagic Freeform Plus V2022.0.34, Hexagon AB, Stockholm, Sweden) with the implant geometry optimized for each cadaver specimen’s unique orbital anatomy following anatomical landmarks. The 3D-printed PSIs included a native, unfilled medical-grade PEEK (Figure 1B) and a radiopaque BaSO_4_-PEEK formulation containing 20% BaSO_4_ by weight (Figure 1C). Prior to cadaveric surgery, the PEEK PSI fit and positioning were verified using the corresponding anatomical models (Figure 2A).

Fabrication was performed using a material extrusion 3D printer (EXT 220 MED, 3D Systems, Rock Hill, SC, USA). A layer height of 0.26 mm was applied, and implants were 3D-printed with a wall thickness of 0.8 mm. Extended support structures surrounding each implant were employed to ensure printability and to increase the nozzle travel distance between layers, thereby preventing thermal build-up and associated layer collapse. Post-processing comprised support removal and surface refinement to facilitate surgical handling. Drainage holes were manually introduced during post-processing rather than generated in the printed geometry.

### 2.4. Surgical Technique and Implant Placement

Standardized surgical approaches were employed for all procedures to ensure consistent evaluation conditions. A transconjunctival incision with lateral canthotomy was used to access the orbital floor. For reconstructions requiring medial wall access, a transcaruncular approach was additionally utilized. Following periosteal incision and subperiosteal dissection, the orbital floor and walls were exposed to simulate appropriate fracture defect sites according to the assigned complexity classification. Physical fractures were not created in the cadaveric specimens. Instead, implants were placed on intact orbital anatomy following virtual surgical planning, using it as a reconstruction-to-native-position simulation. This approach enabled the standardized evaluation of implant handling, fit perception, and workflow efficiency across all specimens.

The pre-fabricated titanium mesh implants underwent intraoperative manual contouring and trimming to achieve an appropriate anatomical fit. Pre-bent meshes, initially shaped using 3D-printed anatomical models, required additional chairside adjustments, including cutting and bending, to achieve optimal defect coverage. The intraoperative bending time was recorded from initiation to completion of the final contouring, while the 3D-printed PEEK PSIs were oriented using anatomical landmarks established during virtual surgical planning, including the inferior orbital rim, the inferomedial orbital strut, and the transition zone between the orbital floor and the medial wall. Each PSI design incorporated reference points corresponding to these landmarks, and positioning was verified against the 3D-printed anatomical biomodels prior to and during implant placement. All implants were secured using the standardized fixation protocols appropriate for each material type. Titanium meshes and PSIs were secured using 1.5 mm diameter, 6 mm length SpeedTip surgical screws from the Medartis MODUS 2 Midface fixation system (Medartis AG, Basel, Switzerland).

### 2.5. Outcome Measures and Data Collection

Primary outcome measures included operative efficiency assessed through objective timing measurements: intraoperative bending time for titanium mesh and total procedure time for all implant types (measured in minutes using standardized timing protocols). Surgical performance was evaluated using a structured 10-question survey instrument administered immediately following each procedure.

The survey assessed six key performance domains using the following standardized 5-point Likert scales: (1) handling characteristics during implant manipulation (1 = easy, 5 = hard), (2) implant fit quality in the final reconstruction (1 = very good, 5 = poor), (3) position confidence regarding correct final implant placement (1 = certain, 5 = uncertain), (4) mechanical stability assessment including concerns about potential implant failure (1 = very stable, 5 = unstable), (5) learning curve requirements for mastering implant techniques (1 = easy, 5 = hard), and (6) insertion confidence reflecting ease of implant placement technique (1 = difficult, 5 = easy).

Secondary outcomes included the ability to distinguish between native (standard) and BaSO_4_-enhanced PEEK PSIs based solely on tactile feedback (1 = clear difference, 5 = indistinguishable), the overall preference when given a free choice between implant options, and the identification of procedural challenges through open-ended responses. Fourteen CMF surgical team members utilized 18 operative sites across 9 cadaver heads. Each participant performed one or two procedures according to predefined specimen allocation and logistical availability. A material differentiation assessment was conducted immediately following each PEEK PSI procedure, with the surgeons blinded to the specific formulation throughout the evaluation. All evaluations were completed independently by each surgeon immediately post-procedure to minimize recall bias, with data collection supervised by trained research personnel using standardized protocols to ensure consistent administration across all participants.

### 2.6. Statistical Analysis

The sample size of 9 cadaveric heads (18 orbital sites) was determined by specimen availability within the study timeframe, consistent with established cadaveric studies in orbital reconstruction research utilizing comparable sample sizes. Given the exploratory nature of this comparative technology evaluation, a formal power analysis was not conducted.

The statistical analysis emphasized descriptive statistics and effect size estimation to assess the clinical meaningfulness of observed differences. Continuous variables were reported as mean ± standard deviation, and categorical variables were reported as frequencies and percentages. Cohen’s d effect sizes were calculated to quantify the magnitude of differences between implant types, with interpretations following established conventions (small: 0.2–0.5; medium: 0.5–0.8; large: >0.8). This approach was selected as appropriate for the exploratory study objectives, emphasizing clinical significance over statistical significance testing. The learning curve assessment was based on the surgeons’ perceived procedural difficulty following limited exposure (1–2 procedures). A sensitivity analysis was performed, excluding the medical student and senior surgeon, to assess the impact of experience-level outliers.

## 3. Results

### 3.1. Participant Demographics

Fourteen CMF surgical team members participated in the evaluation and provided complete datasets for analysis. The cohort included 12 male (85.7%) and 2 female (14.3%) surgeons with a mean age of 33.6 ± 7.0 years (range: 24–49 years). The experience levels comprised ten residents (71.4%), two attending physicians (14.3%), one senior physician (7.1%), and one medical student (7.1%). The number of years of surgical experience ranged from 0 to 22 years, with the majority of participants being early-career surgeons. Nine participants (64.3%) reported no prior orbital reconstruction experience. These participants received standardized orientation on surgical approaches and implant handling principles prior to evaluation. The detailed participant demographics are summarized in Table 2. 

To evaluate the potential influence of experience-level outliers on surgeon-reported outcomes, a sensitivity analysis was performed, excluding the medical student and the senior surgeon. A comparative evaluation of operative time, handling, fit quality, positioning confidence, mechanical stability, and learning curve perception showed no meaningful change in the observed trends across the implant types. The relative performance patterns remained consistent with those observed in the full cohort.

### 3.2. Operative Efficiency and Time Measurements

The pre-fabricated titanium meshes’ intraoperative bending time averaged 6.1 ± 5.6 min. The complete titanium mesh workflow (pre-bending and insertion) time was 11.2 ± 5.1 min compared to 9.5 ± 5.3 min for the 3D-printed PEEK PSIs, representing a 1.6-min reduction (14.7% decrease) in total procedure time (Cohen’s d = −0.30, small effect).

### 3.3. Surgical Performance Domain Characteristics

A comparative surgeon assessment across the six performance domains (Table 3) revealed the advantages of the 3D-printed PEEK PSIs in handling, fit quality, and mechanical stability, while the titanium mesh demonstrated easier learning curve requirements (Cohen’s d = 0.52, medium effect). Most differences were small in magnitude, with positioning and insertion confidence showing minimal variation between the implant types.

An analysis of the individual surgeon responses revealed varying degrees of consensus across performance domains. For handling characteristics, both implant types showed similar response ranges (Titanium: 2–4, PEEK: 1–4), with 73% of the titanium mesh assessments and 67% of PEEK assessments rated as 2–3 (easy to moderate handling). Fit quality assessments demonstrated notable variability for the PEEK PSIs (range 1–4), with 58% of responses rated as 1–2 (very good to good) compared to the titanium mesh showing more consistent ratings (range 1–3) with 64% rated as 2 (good), reflecting both fracture complexity differences and patient-specific design precision.

Mechanical stability assessments revealed the strongest consensus favoring the PEEK PSIs, with 100% of the PEEK ratings at 1–2 (very stable to stable) compared to 91% for the titanium mesh, demonstrating clear surgeon confidence in patient-specific implant structural integrity. Position confidence assessments (certainty of correct final positioning) showed similar patterns across both materials (ranging from 1–4), with 67% of the PEEK assessments and 64% of the titanium mesh assessments rated 1–2 (certain to mostly certain), indicating comparable surgeon confidence in final implant positioning regardless of technology.

Learning curve assessments confirmed the expected complexity differential, with the PEEK PSIs consistently rated as more difficult to learn (range 3–5, no ratings below 3) compared to the titanium mesh (range 2–5, with 18% rated as 2), reflecting the inherent complexity of Class IV reconstructions versus simpler fracture patterns. Insertion confidence assessments showed considerable variability for both materials, with the PEEK PSIs demonstrating the widest response distribution (range 1–5) compared to the titanium mesh (range 2–5), indicating that surgeon comfort levels with patient-specific insertion techniques varied among the participants.

### 3.4. Material Differentiation and Surgeon Preferences

The surgeons demonstrated limited ability to distinguish between the standard PEEK and radiopaque BaSO_4_-PEEK PSIs during surgical procedures, with a mean differentiation score of 4.20 ± 1.40 on the 5-point scale (1 = clear difference, 5 = indistinguishable). When given a free choice among the implant options, the surgeon’s preferences were notably situation-dependent. Most respondents (9/14, 64.3%) indicated that their preference would depend on the specific clinical situation, while two surgeons (14.3%) preferred the 3D-printed PEEK PSIs, one surgeon (7.1%) preferred the titanium mesh, and one surgeon (7.1%) expressed no preference.

### 3.5. Surgeon-Reported Technical Challenges

Open-ended responses regarding potential surgical difficulties revealed distinct challenge patterns between the implant types. For the pre-fabricated titanium mesh implants, the most frequently cited concerns included intraoperative shape instability during insertion (4/11 respondents, 36%), requirement for extensive bone exposure (3/11, 27%), and contouring difficulties (3/11, 27%). Additional titanium mesh-related challenges included sharp edges during handling (2/11, 18%) and concerns about implant positioning accuracy (2/11, 18%).

On the other hand, the 3D-printed PEEK PSI-related challenges focused primarily on implant thickness perception (3/12 respondents, 25%), planning software learning requirements (2/12, 17%), and occasional dimensional sizing concerns (2/12, 17%). Several surgeons noted no specific technical difficulties with either implant type (Titanium: 2/11; PEEK: 4/12).

Representative surgeon comments included the following: “Mesh doesn’t hold its shape during insertion” and “You have to expose a lot of bone to insert the mesh” for titanium-related concerns; while the 3D-printed PEEK PSI feedback included the following: “Feels thick compared to titanium mesh” and “Need time for planning and knowledge of using the software.”

### 3.6. Experience-Stratified Analysis

To evaluate the potential influence of surgical experience on primary outcomes, the participants were stratified into trainees (*n* = 10) and attending/senior surgeons (*n* = 4). The experience-stratified analysis is presented in Table 4.

Comparable directional trends were observed across the experience strata for operative time, handling characteristics, fit quality, positioning confidence, mechanical stability, and learning curve perception for both the titanium mesh and the 3D-printed PEEK PSIs. The trainees demonstrated slightly shorter procedure times for both the titanium mesh (10.4 ± 4.9 min) and the PEEK PSIs (9.1 ± 5.0 min) compared to the attending/senior surgeons (13.5 ± 5.8 min and 10.4 ± 5.9 min, respectively). The perceived learning curve was marginally steeper among trainees for the PEEK PSIs (3.6 ± 0.7) than among attending/senior surgeons (3.4 ± 0.6), reflecting expected differences in familiarity with virtual surgical planning workflows. Overall, experience stratification did not materially alter the relative performance trends between implant types.

## 4. Discussion

This cadaveric evaluation demonstrates that the 3D-printed PEEK PSIs and titanium mesh each offer distinct advantages for orbital reconstruction depending on case complexity and clinical priorities. The predominantly trainee-dominant population provides valuable insights into technology adoption patterns among future practitioners while offering a unique perspective on learning curve requirements and technology preferences among surgeons who will drive future clinical practice. Despite limited clinical experience, the finding that 64.3% of the participants preferred a situation-dependent implant selection demonstrates the clinical reasoning and understanding of individualized treatment approaches.

The 14.7% reduction in total procedure time with the 3D-printed PEEK PSIs represents a clinically meaningful improvement in surgical efficiency, consistent with previous studies demonstrating that PSIs and computer-assisted workflows can reduce intraoperative time and streamline surgical procedures in orbital reconstruction [15]. A recent meta-analysis of 564 patients showed that pre-shaped implants on 3D-printed anatomical models reduced operative time by 23.58 min compared to manual free-hand shaping [16], while Chepurnyi et al. reported an approximately 34% reduction in surgical time with PEEK PSIs compared to pre-bent titanium plates in orbital reconstruction [17]. The elimination of intraoperative bending procedures directly addresses a primary limitation of conventional titanium mesh reconstruction. Our finding that bending titanium mesh alone required an average of 6.1 min underscores the inefficiency of intraoperative adaptation, particularly in complex cases, where PEEK PSI technology eliminates this step through precise virtual surgical planning.

The 3D-printed PEEK PSIs demonstrated advantages in handling, fit quality, and mechanical stability, with mechanical stability showing the most clinically meaningful difference (Cohen’s d = −0.47). The unanimous rating of the 3D-printed PEEK PSIs as stable to very stable (100% rated 1–2), compared with the 91% rating for the titanium mesh, reflects the surgeons’ confidence in the PSIs’ structural integrity. These advantages reflect the precision afforded by a patient-specific design, which is crucial for multi-walled, complex orbital fractures, where reduced surgeon concern about implant failure during positioning is critical in limited-access regions [18,19]. Previous comparative studies have demonstrated superior volumetric restoration with PSIs, with mean orbital shape differences between reconstructed and intact orbits of 0.137 cm^3^ for PSIs versus 1.05 cm^3^ for conventional plates, particularly in complex reconstructions requiring cantilevered support [20].

The surgeon-reported technical challenges provide an important context for quantitative assessments and highlight technology-specific implementation considerations. Titanium mesh concerns are centered on intraoperative handling issues, including shape instability during insertion, extensive bone exposure requirements, and contouring difficulties. Such challenges are consistent with previous cadaveric evaluations, demonstrating the inherent limitations of manual intraoperative adaptation despite pre-bending on anatomical models [21]. By contrast, the 3D-printed PEEK PSI challenges are focused on implant thickness perception and the learning requirements for planning software. These distinct challenge patterns support the complementary nature of these technologies and inform targeted training protocols for successful implementation.

Pre-bending of the titanium mesh on 3D-printed anatomical models demonstrated easier learning curve requirements compared to the 3D-printed PEEK PSIs. However, this finding must be interpreted within the fracture complexity context rather than purely material properties. The 3D-printed PEEK PSIs were evaluated in complex Class IV orbital fracture scenarios, while the titanium mesh was assessed in simpler Class II defects with preserved anatomical landmarks. The perceived increased difficulty of the 3D-printed PEEK PSI procedures likely reflects the inherent complexity of reconstructions rather than material-handling characteristics alone, consistent with studies demonstrating rapid skill acquisition with virtual surgical planning technologies, in which novice users achieved proficiency within six planning sessions, with significant reductions in both planning time and accuracy [22]. This suggests that implant selection should be matched to both the surgeon’s experience and the case’s complexity.

Stratification of primary outcomes by level of surgical experience showed comparable directional trends between trainees and attending/senior surgeons for both titanium mesh and 3D-printed PEEK PSIs. While experienced surgeons showed slightly higher procedural confidence and marginally longer procedure times, relative implant performance across handling, fit quality, positioning confidence, mechanical stability, and learning curve perception remained consistent across the experience strata. These findings suggest that the observed differences among implant technologies were not driven solely by operator experience and support the generalizability of the results across a broad range of training levels.

The successful development and validation of radiopaque PEEK formulations could represent a paradigm shift in orbital implant technology, addressing a fundamental limitation that has historically limited PEEK’s use in orbital reconstruction. PEEK’s traditional radiolucency has created a clinical dilemma where the postoperative visualization of implant position is essential for detecting complications such as implant migration or soft tissue entrapment. BaSO_4_ has emerged as the preferred radiopaque filler for high-temperature polymers, like PEEK, due to its thermal stability at processing temperatures exceeding 335 °C, its excellent biocompatibility, and its ability to provide radiographic contrast without significant artefact formation. These properties distinguish it from alternative radiopaque additives, such as bismuth compounds, which degrade at lower temperatures [23]. The incorporation of BaSO_4_ at optimal concentrations achieves controlled radiopacity and is expected to facilitate precise implant localization on standard CT images while preserving superior soft tissue contrast compared to metallic alternatives. The seamless integration of radiopaque enhancement without detectable changes in handling characteristics validates the potential for broader clinical adoption without requiring modifications to established surgical techniques.

The incorporation of barium sulfate into PEEK raises important considerations regarding cytotoxicity and long-term matrix stability. BaSO_4_ is a chemically inert, insoluble, and thermally stable radiopaque additive that has been widely used in implantable polymeric biomaterials for decades. It has an extremely low aqueous solubility which prevents ion release and associated cellular toxicity, and preclinical testing has demonstrated that BaSO_4_-filled PEEK exhibits cytocompatibility comparable to native PEEK, meeting ISO 10993 biocompatibility standards for implantable devices [12]. Regarding concerns of filler disintegration, BaSO_4_ particles are homogeneously dispersed within the PEEK matrix during high-temperature compounding (>350 °C), forming a stable composite structure. At the 20% filler concentration used in this study, within the established 10–30% range for medical applications, matrix integrity is maintained under physiological loading conditions, with no evidence of particulate release reported in mechanical testing. Nevertheless, long-term clinical studies specific to orbital patient-specific implants remain warranted to confirm these favorable preclinical findings [24,25]. Table 5 summarizes the comparative advantages and limitations of each orbital reconstruction material evaluated in this study.

The situation-dependent preferences expressed by the trainee surgeons provide encouraging insights into contemporary surgical education and clinical decision-making. This pattern suggests recognition that optimal implant selection should be individualized based on defect complexity, anatomical requirements, surgeon experience, and available resources. Our findings support a nuanced approach to orbital implant selection where titanium mesh remains appropriate for simple fractures where manual contouring is feasible and cost-effectiveness is prioritized, while 3D-printed PEEK PSIs offer advantages for complex multi-walled fractures requiring precise anatomical restoration and improved imaging compatibility.

Several considerations should guide the interpretation of these findings. First, the study design intentionally matched implant types to clinically appropriate fracture complexities, reflecting real-world surgical decision-making rather than a controlled material comparison within identical defect patterns. While this approach provides realistic evaluation scenarios, it precludes a direct comparison of intrinsic material properties under identical anatomical conditions and is therefore acknowledged as a limitation of the study. Second, physical fractures were not created in the cadaveric specimens; implant placement was performed on intact orbital anatomy following reconstruction-to-native-position simulation, while these standardized evaluation conditions, fracture-specific biomechanics, and soft-tissue behavior were not replicated.

Third, the participant cohort was heterogeneous, with a predominance of trainees. The heterogeneous experience levels, including one medical student and one senior surgeon at opposite ends of the training spectrum, may introduce variability. However, the inclusion of one medical student enabled an assessment of the baseline intuitiveness of the workflow for a novice user. A sensitivity analysis excluding these experience-level outliers demonstrated no impact on the overall observed trends. The results should therefore be interpreted as reflecting perceptions of the technology across a range of training levels rather than expert consensus. The trainee-dominant cohort provides valuable insights into technology adoption patterns and learning curve requirements among future practitioners, although incorporation of additional experienced surgeon perspectives would further strengthen generalizability. Furthermore, the learning curve assessment was based on perceived difficulty following limited exposure (1–2 procedures) rather than longitudinal skill acquisition. True learning curve characterization would require prospective evaluation with repeated procedures.

Lastly, the cadaveric model offers controlled evaluation conditions while recognizing that live tissue properties, healing responses, and patient-specific factors contribute additional complexity to clinical outcomes. While volumetric and technical outcomes favor PSIs, systematic reviews have indicated that clinical outcomes remain variably reported across studies, with current evidence limited to level III, highlighting the need for prospective comparative trials with standardized outcome measures [26].

While patient-specific titanium implants represent an important emerging technology, their availability remains limited to highly specialized centers. By contrast, pre-fabricated titanium mesh remains the global standard of care for orbital reconstruction. This study, therefore, focused on a clinically relevant decision point faced by most surgeons: whether to adopt point-of-care polymer patient-specific implants or to continue conventional titanium mesh workflows. A comparative evaluation of patient-specific titanium versus patient-specific polymer implants is an important direction for future research. Future research should focus on clinical trials that compare outcomes within matched fracture-complexity categories, incorporating experienced surgeon perspectives alongside trainee evaluations. Cost-effectiveness analyses considering case complexity factors, learning curves, and long-term outcomes would provide valuable guidance for institutional technology adoption decisions. An investigation of structured training protocols for implementing 3D-printed PEEK PSIs, along with studies of long-term clinical outcomes, would provide essential data for evidence-based practice guidelines.

## 5. Conclusions

This cadaveric evaluation demonstrates that the 3D-printed PEEK PSIs and pre-fabricated titanium mesh implants represent complementary technologies in orbital reconstruction, with each offering distinct advantages for specific clinical scenarios. Within clinically appropriate fracture–implant matching scenarios, the 3D-printed PEEK PSIs demonstrated advantages in handling, fit quality, and mechanical stability for complex Class IV defects, while the pre-bent titanium mesh showed a lower learning curve for Class II reconstructions. These findings support situation-dependent implant selection aligned with current clinical practice patterns. The successful development of the radiopaque PEEK formulations addresses a critical limitation in orbital applications, potentially facilitating postoperative surveillance without compromising handling characteristics. The situation-dependent preferences expressed by surgeons support personalized technology selection based on case complexity and clinical priorities, advancing orbital reconstruction toward precision medicine approaches that optimize outcomes through appropriate technology matching.

## Figures and Tables

**Table 1:** Orbital fracture classification used in this study.

Class	Definition	Surgical Complexity	Implant Type Evaluated
Class II	Defects > 2 cm^2^ of orbital floor and/or medial wall within zones 1–2	Moderate	Pre-bent titanium mesh
Class IV	Entire orbital floor and medial wall defects extending into posterior third (zone 3) with missing bony ledge	Complex multi-walled reconstruction	3D-printed PEEK PSI (native and with BaSO_4_)

**Table 2:** Participant Demographics and Surgical Experience.

Parameter	Value
Number of participants	14
Sex (male/female)	12/2
Age (years)	33.6 ± 7.0 (range 24–49)
Experience level	
Residents	10 (71.4%)
Attending physicians	2 (14.3%)
Senior physician	1 (7.1%)
Medical student	1 (7.1%)
Years of surgical experience	0–22 years
Prior orbital reconstructions	
None	9 (64.3%)
1–10 cases	2 (14.3%)
>10 cases	3 (21.4%)

**Table 3:** Comparative surgical performance assessment: Titanium Mesh and 3D-Printed Polyetheretherketone (PEEK) patient-specific orbital implants.

Domain	Ti Mesh	3D-Printed PEEK PSI	Cohen’s d	Advantage
Handling (1 = easy, 5 = hard)	2.82 ± 0.75	2.75 ± 0.97	−0.08	PSI (PEEK)
Fit Quality (1 = very good, 5 = poor)	2.18 ± 0.60	2.00 ± 1.04	−0.21	PSI (PEEK)
Position Confidence (1 = certain, 5 = uncertain)	2.27 ± 1.01	2.25 ± 1.06	−0.02	Similar
Stability (1 = stable, 5 = unstable)	1.91 ± 0.54	1.67 ± 0.49	−0.47	PSI (PEEK)
Learning (1 = easy, 5 = hard)	3.18 ± 0.87	3.58 ± 0.67	0.52	Pre-bent (Ti)
Insertion Confidence (1 = difficult, 5 = easy)	3.36 ± 0.92	3.25 ± 1.14	−0.11	Similar

Surgeon ratings (mean ± SD) across performance domains using 5-point Likert scales. Lower scores indicate better performance except for Confidence (1 = difficult, 5 = easy). Cohen’s d: effect size magnitude. Titanium (Ti), polyetheretherketone (PEEK), patient-specific implant (PSI).

**Table 4:** Primary Outcomes Stratified by Level of Surgical Experience.

Outcome Measure	Implant	Trainees (*n* = 10) Mean ± SD	Attending/Senior (*n* = 4) Mean ± SD
Procedure Time (min)	Titanium Mesh	10.4 ± 4.9	13.5 ± 5.8
	3D-Printed PEEK PSI	9.1 ± 5.0	10.4 ± 5.9
Handling (1 = easy, 5 = hard)	Titanium Mesh	2.8 ± 0.8	2.9 ± 0.7
	3D-Printed PEEK PSI	2.7 ± 1.0	2.8 ± 0.9
Fit Quality (1 = very good, 5 = poor)	Titanium Mesh	2.2 ± 0.6	2.1 ± 0.5
	3D-Printed PEEK PSI	2.0 ± 1.1	1.9 ± 0.8
Position Confidence (1 = certain, 5 = uncertain)	Titanium Mesh	2.3 ± 1.0	2.2 ± 0.9
	3D-Printed PEEK PSI	2.3 ± 1.1	2.2 ± 1.0
Mechanical Stability (1 = stable, 5 = unstable)	Titanium Mesh	1.9 ± 0.6	1.8 ± 0.5
	3D-Printed PEEK PSI	1.7 ± 0.5	1.6 ± 0.5
Learning Curve (1 = easy, 5 = hard)	Titanium Mesh	3.2 ± 0.9	3.1 ± 0.8
	3D-Printed PEEK PSI	3.6 ± 0.7	3.4 ± 0.6

Lower scores indicate better performance except where stated.

**Table 5:** Comparative advantages and limitations of orbital reconstruction materials.

Parameter	Ti Mesh	3D-Printed PEEK PSI (Native)	3D-Printed PEEK PSI (BaSO_4_)
Advantages			
Radiopacity	Inherent	None	Controlled
Imaging compatibility	Limited	Excellent	Good
Intraoperative adaptability	High	Low	Low
Anatomical precision	Surgeon-dependent	High (patient-specific)	High (patient-specific)
Clinical experience	Extensive (>30 yrs)	Growing	Emerging
Limitations			
CT/MRI artifacts	Significant	None	Minimal
Sharp edge risk	Present	Absent	Absent
Planning requirements	Minimal	VSP required	VSP required
Lead time	Immediate	Days-weeks	Days-weeks

## Data Availability

The datasets of this study are available from the corresponding author upon reasonable request.

## Abbreviations

The following abbreviations are used in this manuscript:

3D	Three-Dimensional
AM	Additive Manufacturing
BaSO_4_	Barium Sulfate
CAD	Computer-Aided Design
CMF	Craniomaxillofacial Surgery
CT	Computed Tomography
FFF	Fused Filament Fabrication
PEEK	Polyetheretherketone
PLA	Polylactic Acid
PSI	Patient-Specific Implant
SD	Standard Deviation
Ti	Titanium
